# Improved brain tumor diagnostics and follow-up with novel magnetic resonance imaging methods: A single center study protocol

**DOI:** 10.1371/journal.pone.0336387

**Published:** 2025-11-14

**Authors:** Jesse Lohela, Kaisa Lehtiö, Kalle Inget, Sakari S. Karhula, Susanna Piironen, Angélica Suutari, Antti Knuutinen, Miro Jänkälä, Eveliina Lammentausta, Michaela K. Bode, Juha Nikkinen, Niina Salokorpi, Tuija Keinänen

**Affiliations:** 1 Department of Oncology and Radiotherapy, Oulu University Hospital, Oulu, Finland; 2 Research Unit of Health Sciences and Technology, University of Oulu, Oulu, Finland; 3 Medical Research Center Oulu, Oulu University Hospital and University of Oulu, Oulu, Finland; 4 Neurocenter, Oulu University Hospital, Oulu, Finland; 5 Department of Diagnostic Radiology, Oulu University Hospital, Oulu, Finland; Goethe University Hospital Frankfurt, GERMANY

## Abstract

This protocol outlines a prospective study aimed at enhancing the diagnosis and monitoring of brain tumors through advanced non-invasive imaging techniques. While magnetic resonance imaging (MRI) is a cornerstone of brain tumor diagnostics, it often lacks the specificity required for definitive diagnosis, which typically relies on invasive tissue sampling. To address this, the study will evaluate advanced MRI techniques—such as perfusion, diffusion, blood-oxygen-level-dependent imaging, magnetic resonance spectroscopy, and amide proton transfer-weighted imaging— that offer valuable physiological and molecular insights, beyond conventional anatomical imaging. Despite their potential, clinical adoption of these methods remains limited. MRI also plays a central role in treatment response assessment and follow-up, yet conventional anatomical sequences may not detect early physiological changes or differentiate true progression from pseudoprogression. Advanced imaging methods have shown promise in addressing these limitations, and predictive models for recurrence risk could further personalize treatment strategies. In this study, imaging will be performed using a standardized 3T MRI scanner at multiple time points: preoperatively, before radiotherapy, during treatment, and throughout follow-up. This protocol aims to establish a multiparametric imaging framework capable of capturing dynamic physiological and molecular changes in brain tumors. The primary goal is to determine whether combining advanced sequences improves diagnostic accuracy compared to conventional MRI, using histopathology as the reference. Secondary objectives include predicting treatment response, distinguishing true progression from pseudoprogression, and modeling spatial recurrence risk based on quantitative imaging biomarkers. We hypothesize that a multiparametric imaging approach will, enable earlier detection of tumor progression and support more precise, individualized treatment decisions.

## Introduction

Diagnostics and characterization of brain tumors are done on the basis of either magnetic resonance imaging (MRI) or a sample taken from the tumor [1]. In majority of cases, MRI cannot give the exact diagnosis, and an accurate histological diagnosis can only be determined from the tissue sample. Obtaining invasive tumor samples either with biopsy or in open surgery has notable risks [2]. Hence, there is a need for a reliable, non-invasive detection method to identify brain tumor types and malignancies safely.

The treatment plan is usually made based on the diagnosis obtained from the MRI and tissue samples. Thus, accurate diagnosis is crucial for patients’ further treatment. Treatment strategies vary depending on the tumor type, grading, patient age, clinical condition, including functional level, and molecular characteristics of the tumor [3,4]. In most cases, surgical resection is the primary treatment option. Radiotherapy is utilized on unreachable targets or as an adjuvant treatment after surgical resection [4]. Chemotherapeutic agents can be combined with radiotherapy or utilized as the main treatment modality in certain cases [5].

To improve tumor diagnostics, more advanced imaging sequences are required. Development of novel MRI devices and sequences has reduced the imaging time significantly, making them more feasible in clinical imaging settings as well. Perfusion imaging can be used to evaluate the tumor grading, as higher-grade tumors have different microvascularity compared to lower grade tumors. Additionally, diffusion imaging and blood-oxygen level dependent (BOLD) imaging have shown potential for tumor grading [6–9] but neither of these are yet widespread in clinical use. Even though these methods can give some information about tumor grading, they are not specific to different tumor subtypes, and they all fail to provide information about the molecular characteristics of the particular tumor.

The molecular content of the tumor can be studied using magnetic resonance spectroscopy (MRS) and chemical exchange saturation transfer (CEST) imaging methods. Among the CEST methods, especially the amide proton transfer weighted (APTw) imaging has shown promising results [10–12]. Both methods, MRS and APTw, provide complementary molecular-level information beyond conventional water proton signals, thereby offering insights that are not attainable with standard MRI techniques. Although these techniques hold promise for enhancing the diagnostic accuracy of brain tumors, their clinical implementation remains limited. This is primarily due to variability in imaging analysis outcomes, which can arise from differences in MRI hardware, magnetic field strengths, and acquisition parameters [13,14].

MRI is also the gold standard in the evaluation of current tumor load and follow-up of treatment response [15–17]. The use of Response Assessment of Neuro-Oncology (RANO) criteria and similar MRI protocols are recommended for accurate and comparable assessment of tumor response [1,18–20]. Current recommendations for follow-up imaging include widely used anatomical MRI sequences that do not provide information on the physiological and biological characteristics of the particular tumor. This may lead to a delay in the recognition and treatment of tumor progression.

Advanced imaging methods may provide additional information on treatment response. For example, perfusion imaging can show changes in oxygen status [21], blood volume [22], and perfusion speed [23] in the treatment area. Diffusion imaging can be used to monitor tissue cellular density and differences in microstructures [24], and BOLD can point out hypoxic zones and changes in functional brain areas. [25]. Post-treatment metabolic changes can be studied with MRS and CEST imaging [24,26]. For example, the overall CEST signal decrement has been found to be an early indicator of treatment response [26,27].

Additionally, it is well known that radiotherapy may induce changes in the tumor, such as necrosis and an inflammatory reaction. These conditions can mimic tumor progression (so-called pseudoprogression) on the conventional clinical MRI sequences, which cannot differentiate real progression from pseudoprogression. A more reliable differentiation would improve patient care significantly since treatment choices in these conditions differ greatly from those of real tumor progression. False interpretation of imaging results can lead to unnecessary surgeries, which always include risks and decrease patients’ quality of life, at least temporarily. Currently, there are no clinically relevant methods to differentiate pseudoprogression from tumor progression, even though several previously introduced MRI sequences have shown evidence of increased accuracy [27–34].

For treatment optimization, it would be valuable to be able to predict the location of increased risk for tumor recurrence and/or progression. Such predictive capability would allow for targeted dose escalation in high-risk areas to prevent progression, while enabling dose de-escalation in regions with a lower likelihood of recurrence, thereby minimizing toxicity. Although some promising methods have been proposed, further research is required to improve the accuracy and clinical applicability of spatial risk prediction models [35–37].

The difficulties in diagnostics and follow-up of the heterogenic continuum of brain tumors with traditional MRI are well known, and many attempts have been made to overcome these. Despite numerous studies exploring individual advanced imaging techniques, most have been limited to one or two modalities and lack integration into routine practice. A comprehensive, multiparametric approach is needed to identify optimal combinations of sequences that can support precise diagnosis and treatment decisions throughout the disease course [21–33]. This single-center protocol addresses these gaps by implementing a standardized multiparametric MRI framework using a consistent 3T scanner and harmonized acquisition parameters. The controlled setting minimizes technical variability and enables high-quality, reproducible data collection. Integration with clinical workflows allows real-time validation against histopathological findings and treatment outcomes.

The study combines multiple advanced imaging modalities in a longitudinal design, capturing tumor dynamics across key treatment phases. This approach enables robust evaluation of diagnostic accuracy, prediction of treatment response, differentiation of true progression from pseudoprogression, and modeling of spatial recurrence risk. The findings are expected to inform future multicenter trials and contribute to the development of clinically applicable imaging biomarkers for personalized neuro-oncology care.

## Materials and methods

**Fig 1:**
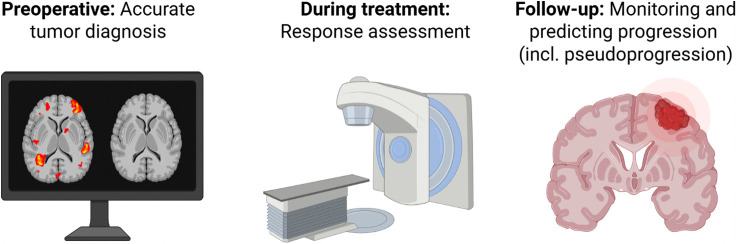
Main objectives of the study: Radiological assessment is integrated throughout the treatment timeline—starting with the preoperative phase, where imaging is used to predict underlying pathology; continuing through oncological treatment, where therapeutic response is evaluated; and extending into the follow-up phase, where imaging aids in assessing and predicting disease progression, including the differentiation of true progression from pseudoprogression. Figure created with BioRender.com (license to publish obtained).

This study protocol was developed in accordance with the SPIRIT (Standard Protocol Items: Recommendations for Interventional Trials) guidelines, adapted as appropriate for a non-randomized design [38]. The aim of this study (Fig 1) is to improve the diagnostics and follow-up of brain tumors using advanced non-invasive multi-parametric MR imaging (mpMRI). All adult brain tumor patients undergoing brain surgery, radiotherapy, or chemotherapy treatment in the Oulu University Hospital (OUH) fulfilling study criteria will be asked to participate in the study. All participants will have MR imaging done using the same 3T MAGNETOM Vida (Siemens Healthineers AG, Forchheim, Germany) at every time point during the treatment course. Current clinical MRI sequences will be obtained according to the usual institutional protocol. Additionally, mpMRI will be performed preoperatively, prior to the radiotherapy, and during the follow-up. Follow-up mpMRI will be performed at the same time as clinical imaging follow-up in accordance with patients’ treatment plans. Participants having radiotherapy will also be scanned midway through the radiation treatment course and after its completion. The number of follow-up scans for each participant will depend on their treatment plans; glioma patients’ follow-up will continue as long as the study is ongoing, and other brain tumors will have at least one one-year follow-up scan after the treatment if no further treatments are planned.

The study began at the end of 2022 following approval by the Ethical Committee of the Northern Ostrobothnia Hospital District. Patient recruitment and data collection are an ongoing process, initially started on March 23, 2023. These phases are expected to be completed by the end of 2027, as the ethical approval remains valid until October 10, 2027. However, if the number of participants is below expectations, amendments to the research protocol and ethical approvals will be submitted to extend the timeframe for participant recruitment. We aim to recruit 50–70 new patients annually, representing approximately 30–50% of those meeting the inclusion criteria each year. This recruitment target has proven feasible: at the time of manuscript submission, 104 patients have been successfully enrolled, yielding a total of 221 individual imaging sessions. We aim to recruit 50 participants representing major brain tumor subtypes—including glioblastoma, low-grade glioma, and meningioma. This sample size is notably larger than the median (~25) typically reported in neuroimaging studies, enabling robust and reliable subgroup analyses [39]. The final results of the study are expected to be available by the end of 2028, assuming no extensions to recruitment or data collection are required.

### MRI sequences

MpMRI studies are conducted with a 64-channel phased-array head coil. Anatomical isotropic 3D T1 and T2-weighted sequences are imaged with resolution ≤ 1 mm^3^ and Dotarem® (Gd-DOTA; Guerbet, Villepinte, France) is used for contrast enhancement when clinical imaging is performed at the same time. Diffusion tensor imaging (DTI) is imaged with 64 directions and b-values of 0 and 1000. If the patient has trouble staying still, the number of directions will be dropped to 20. For 5-minute resting state BOLD imaging, echo planar imaging technique is used, and 300 volumes are collected. Participants are instructed to lay still with their eyes open and not to think about anything particular. MRS is imaged with intermediate echo time (TE = 135ms) in three directions (3D) always when possible due to the anatomical location of the tumor. 3D MRS is planned with either a T1- or T2-weighted image, depending on the visualization of the tumor. If acquiring 3D MRS is not possible, then 2D or single voxel spectroscopies are used. In single voxel MRS, one voxel is placed to contain the tumor, and other is placed contralateral to the tumor containing healthy brain tissue for comparison. Amide proton transfer weighted (APTw) CEST sequence is scanned with 20 volumes and phases from −4.5 ppm to +4.5 ppm with 0.5 ppm intervals. For perfusion imaging, we utilize pseudo-continuous arterial spin labeling (pCASL) with a labeling duration of 1800 ms and a multi-inversion time technique. The time-of-flight sequence is used for planning the pCASL sequence. Dynamic susceptibility contrast MRI (DSC-MRI) is also performed, when contrast enhancement is used.

### Inclusion criteria

- Age ≥ 18
- Suspected brain tumor undergoing brain surgery, radiotherapy, or chemotherapy treatment at Oulu University Hospital
- The patient tolerates repeat MRI scans without sedation

### Exclusion criteria

- Age < 18
- Previous brain tumor treatments
- 3T MRI contraindicated (e.g., metallic implants, cochlear implants, neurostimulators)
- The patient is unable to provide informed consent

### Workflow

**Fig 2:**
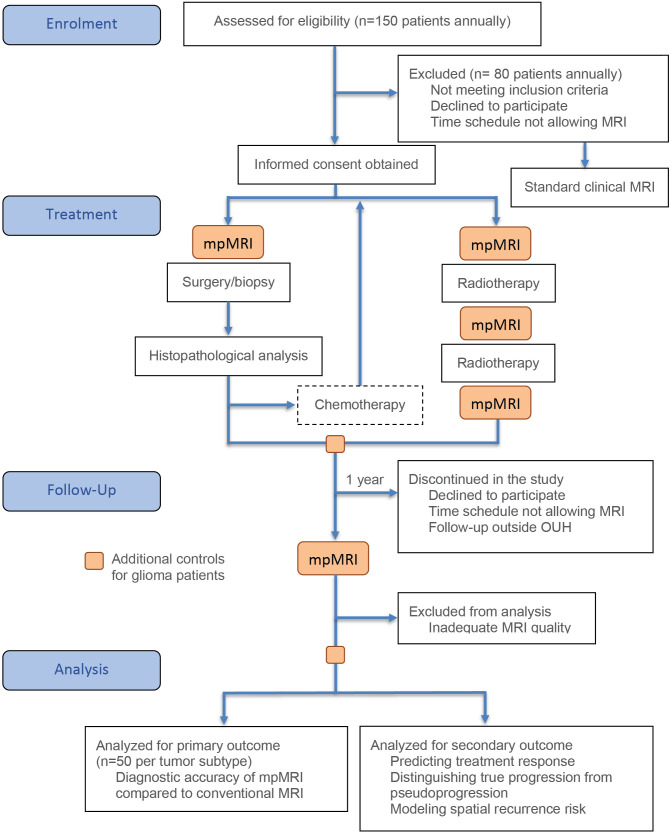
The summary of the study workflow highlights four main phases: enrolment, treatment, follow-up, and analysis. Each phase includes specific tasks such as patient eligibility assessment, application of exclusion criteria, annual recruitment targets, scheduled time points and treatment pathways, as well as primary and secondary analysis endpoints.

The complete workflow of this protocol is illustrated in Fig 2. Once a patient is deemed eligible for the study, the research nurse will seek for oral and written informed consent from the patient for their participation. If the patient agrees to participate, the research nurse schedules an additional mpMRI to be performed alongside the clinically indicated MRI using the 3T MAGNETOM Vida scanner. All study personnel have received training from a medical physicist specialized in MRI, who also provides on-site support during each imaging session. All participants are imaged before the planned treatment. During the surgery, the location from which the sample is taken is either documented using a navigator screenshot or by the surgeon describing the location in the patient’s chart (in the referral letter of the sample for pathology). Radiotherapy planning-related data, including tumor/treatment target and organs at risk contours, simulated radiation dose distributions, and treatment plans, are saved for further evaluation. MpMRI is done before the radiotherapy, midway through the treatment course, and after the last treatment fraction (preferably on the same day). Other follow-up imaging is performed according to the particular patient’s clinical follow-up plan, and at least one one-year follow-up imaging after treatment is taken.

### Data management

The data consists of MR images, radiation treatment plans, analysis data files and clinical information of the patients. All data will be stored on the internal storage platform of Oulu University Hospital, where data is pseudonymized before data analysis. The data is password-protected and can be accessed only by certified members of the project team. The storage and processing of the data complies with the requirements of the Finnish law. When study ends, the principal investigator is responsible of the data destruction. Data sharing and dissemination of study results will follow Finnish legislation and institutional guidelines. Once the results are available, de-identified data may be shared with researchers upon reasonable request and subject to appropriate approvals from relevant authorities. Contact details will be provided in publications presenting the findings.

### Data and statistical analyses

Each patient’s data will be analyzed individually and within different tumor subgroups to study diagnostic accuracy and treatment outcomes. In addition to the research mpMRI protocol, clinical MRI sequences may also be utilized. All MRI data will be thoroughly reviewed following each imaging session, and any scans exhibiting artifacts will be evaluated by a medical physicist with expertise in MRI. To minimize motion-related artifacts, the patient’s head will be stabilized using appropriate supports, and participants will receive clear instructions prior to scanning. When necessary, post-processing motion correction techniques will be applied to address residual artifacts. Datasets with excessive artifacts that compromise image quality will be excluded from analysis. These combined measures are designed to ensure consistent, reliable, and high-quality imaging data throughout the study.

Automatic tumor segmentation will be performed using anatomical T1- and T2-weighted sequences. The possible enhancing tumor area and surrounding edema will be segmented separately. All MRI images will be subjected to image registration to enable automated segmentation also from non-anatomical MRI sequences. For radiotherapy patients, the contours from manual segmentation conducted for the treatment planning will also be used. These include surrounding organs at risk and radiotherapy target structures created and approved by an oncologist.

**Fig 3:**
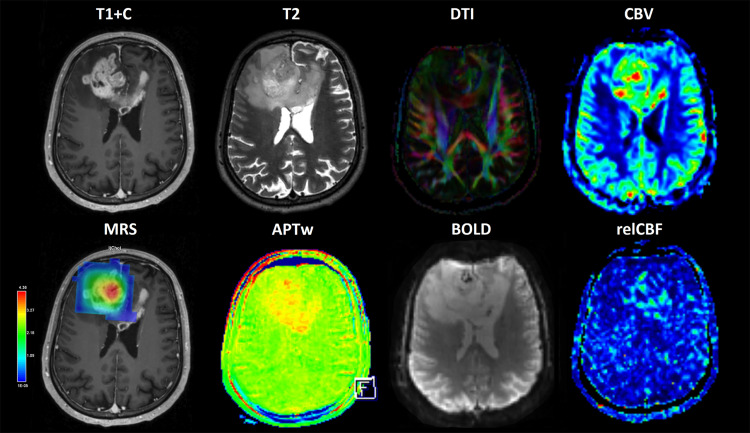
An example of mpMRI findings of a high grade astrocytoma demonstrates the use of both conventional (T1 + C & T2) and more advanced imaging modalities. These include maps derived from diffusion tensor imaging (DTI), cerebral blood volume (CBV), magnetic resonance spectroscopy (MRS), amide proton transfer weighted (APTw) imaging, blood-oxygen level dependent (BOLD) imaging, and relative cerebral blood flow (relCBF). Together, these sequences provide complementary information that enhances diagnostic accuracy and supports a more comprehensive assessment of tumor characteristics.

The composition of different metabolites (choline, N-acetylaspartate, creatine, lactate, and lipide) in the tumor and in its surroundings will be calculated from MR spectroscopy as well as the relations between these. APTw maps will be derived from CEST imaging data using Z-spectrum asymmetry analysis. These maps will then be used to evaluate molecular and metabolic changes in the tissue. From DTI, multiple tensor metrics will be calculated. Maps will be formed for mean, axial, and radial diffusivities, planar, spherical, and linear tensors and for the fractional anisotropy (FA). For perfusion scans (ASL and DSC-MRI), maps of cerebral blood volume (CBV) and relative cerebral blood flow (relCBF) will be calculated. Additional parameters, such as capillary transit-time heterogeneity, oxygen extraction fraction, and cerebral metabolic rate of oxygen, will also be investigated. Various metrics characterizing resting-state functional connectivity will be derived from BOLD imaging data, including independent component analysis and regional homogeneity [40–42]. Examples of different sequences and maps are presented in Fig 3. Radiomics will be analyzed from both segmented tumor regions and normal-appearing brain areas across each MRI sequence. Quantitative imaging features, such as intensity distributions and tissue texture, will be extracted and compared with histopathological findings.

Statistical analyses will be performed at both individual (single-subject) and group levels, accounting for the longitudinal structure of the data. At the individual level, scans from different time points will be co-registered to the pretreatment baseline to enable voxel-wise interpretation. Within-subject changes will be modeled using univariate and multivariate voxel-wise mixed-effects models. Predictors will be standardized and dimensionality reduction (e.g., principal component analysis) applied in cases of strong collinearity. Multiple comparisons will be corrected using false discovery rate or permutation-based threshold-free cluster enhancement within predefined masks (e.g., brain, tumor, or control tissue).

At the group level, subject-specific contrast maps will enter second-level mixed-effects models to test differences across tumor types and clinical subgroups, adjusting for covariates. Cross-sectional comparisons at a given time point will use parametric tests (e.g., t-test, ANOVA) or nonparametric alternatives (e.g., Mann–Whitney U, Kruskal–Wallis) according to distributional assumptions. Multivariable models will evaluate associations between mpMRI features, pathology and clinical outcomes.

Predictive modelling will be evaluated with nested cross-validation and performance metrics (e.g., accuracy, sensitivity, and specificity). Model assumptions will be checked and corrected if needed. Descriptive statistics will summarize patient characteristics and imaging features across treatment phases. Multivariate regression and AI methods will identify tissue-specific patterns and key imaging parameters linked to histopathological outcomes.

Follow-up imaging will be used to study the possible tumor progression and the potential of mpMRI to predict the progression and its location before it can be seen in conventional MR images. Calculated metrics from longitudinal data will also be used to study their potential in treatment efficacy assessment as well as in differentiation between real tumor progression and pseudoprogression. Date and causes of deaths will be requested from the Finnish Official Cause-of-Death Statistics. Overall survival, progression-free survival, and their association with, e.g., mpMRI variables will be analyzed.

### Ethical considerations and declarations

All participants are informed orally and in writing about the study, and a written informed consent is acquired from each subject individually in accordance with the Helsinki Declaration. The study protocol, including all additional imaging methods, has been approved by the Ethical Committee of Northern Ostrobothnia Hospital District, Oulu University Hospital (36/2022). All the data will be pseudonymized prior to the conduct of the analysis.

## Discussion

Brain tumor diagnostics and follow-up have many acknowledged limitations that have not yet been resolved. Many novel imaging sequences have been studied, but none have demonstrated sufficient efficacy alone for widespread clinical application. The continuous development of MRI technology and sequence development now allows faster acquisition times, making it feasible to incorporate multiple advanced imaging sequences alongside standard clinical protocols. We are convinced that for more accurate brain tumor diagnostics, a combination of various novel imaging methods is needed. The same applies also to the evaluation of treatment results and follow-up with early detection of possible progression.

In addition to its role in the diagnosis and follow-up of brain tumors, mpMRI also holds significant value in treatment planning. Advanced imaging sequences can provide detailed information about the extent of tumor infiltration, which can guide surgical planning to maximize tumor resection while preserving critical functional areas identified through functional MRI [43,44]. Meanwhile, integrating the mpMRI approach into clinical workflow has become a point of interest in radiotherapy, potentially leading to more individually tailored treatment. Evidence supporting this includes an introduction of a biologically based targeting method for glioblastoma treatment utilizing the mpMRI approach, as demonstrated by Kim et al. [45,46]. It is clear, that conventional MRI imaging, which is currently mainly used in RT, does not capture all the information available for treatment planning. This highlights the added value of mpMRI for a more personalized treatment strategy.

Although the study aims to demonstrate a potential clinical framework for more personalized treatment strategies, it is important to note that the sequences and institutional practices described are currently implemented only at OUH. In general, many smaller central hospitals, and even some larger university hospitals, may lack the specialized equipment, imaging sequences, or knowledge required to perform such an extensive protocol as applied in this study. This limits the immediate generalizability of the findings, as replication in other institutions may not be feasible without significant technological and procedural adjustments. Nevertheless, by demonstrating the feasibility of these approaches in a single center study design, the study provides valuable insights into currently available clinical imaging solutions with potential to improve patient care and to facilitate broader adoption.

The main limitation with data collection in this study is the wide tertiary catchment area of OUH, which covers approximately half of the land area of Finland. This includes five central care hospitals performing MRI studies. Patients can be referred for treatment at OUH from other hospitals with prior MR imaging performed outside of OUH. In some cases, the surgery may be planned and performed without additional imaging at OUH. Additionally, for some patients, the follow-up imaging can be performed in other hospitals if best suited for the patient. In addition, if a patient undergoes urgent surgery, prior study recruitment and imaging according to the study protocol may not be feasible. The strength of this study is that OUH is the only hospital in the area where neurosurgical procedures or radiotherapy can be performed.

Research involving brain tumor patients presents unique challenges, particularly due to the neurological impact of the disease. One significant issue is the variability in patients’ functional abilities; some individuals may struggle to remain still during extended imaging sessions. This poses a problem since many advanced imaging sequences are susceptible to motion, which can degrade image quality. Although various motion correction techniques exist to mitigate these artifacts [47], in severe cases, the resulting data may still be too distorted to be usable. Another point of consideration is the complexities introduced by postoperative imaging. Foreign materials such as titanium clamps or plates used to close the craniotomy defect can lead to metal artefacts, and the hemostatic material left in the resection cavity can change the MRI signal, causing challenges when evaluating the residual tumor.

Beyond motion- or implant-related issues, novel MRI techniques are highly sensitive to a range of other artifact sources, making image quality monitoring essential for ensuring the reliability of the collected data. Each sequence is reviewed immediately after acquisition, and any observed artifacts or technical issues are documented by the personnel responsible for data collection. These observations are considered during the data analysis phase to account for their potential impact. In addition to visual inspection, routine MRI quality assurance is conducted at daily, monthly, and annual intervals, in accordance with the guidelines of Oulu University Hospital.

Advanced MRI techniques each provide distinct and complementary information; however, no single sequence offers a definitive representation of the underlying neuropathology. The combination of multiple MRI parameters has the potential to yield more reliable and comprehensive diagnostic insights. Technological advancements have made such multi-parametric approaches increasingly feasible. Nonetheless, the routine implementation of these techniques in clinical practice remains constrained – not only by the lack of standardization in both acquisition methods and processing pipelines, but also by software tools that are often complex, time-consuming, and not well-suited for clinical workflows. Through the collaborative efforts of our multidisciplinary team, we aim to address these challenges and facilitate the broader clinical adoption of advanced multiparametric MRI in neuroimaging.

Our mpMRI protocol is applied throughout the entire treatment pathway, offering valuable insights at multiple stages of patient care. The multidisciplinary collaboration between neurosurgery, oncology, radiology, and pathology facilitates seamless information sharing across specialties, supporting the optimization of individualized treatment strategies in the future.
